# Pregnancy-induced changes in serum concentrations of perfluoroalkyl substances and the influence of kidney function

**DOI:** 10.1186/s12940-020-00626-6

**Published:** 2020-07-08

**Authors:** Christel Nielsen, Ulrika Andersson Hall, Christian Lindh, Ulf Ekström, Yiyi Xu, Ying Li, Agneta Holmäng, Kristina Jakobsson

**Affiliations:** 1 Department of Laboratory Medicine, Division of Occupational and Environmental Medicine, Lund University, Medicon Village (402A), Scheelevägen 8, 223 81 Lund, Sweden; 2 Department of Physiology, Institute of Neuroscience and Physiology, Sahlgrenska Academy, University of Gothenburg, Göteborg, Sweden; 3 Department of Laboratory Medicine, Division of Clinical Chemistry and Pharmacology, Lund University, Lund, Sweden; 4 School of Public Health and Community Medicine, Institute of Medicine, Sahlgrenska Academy, University of Gothenburg, Göteborg, Sweden; 5 Occupational and Environmental Medicine, Sahlgrenska University Hospital, Göteborg, Sweden

**Keywords:** Perfluoroalkyl substances, Pregnancy, Glomerular filtration rate, glomerular pore size

## Abstract

**Background:**

Epidemiological associations between maternal concentrations of perfluoroalkyl substances (PFAS) and birth weight are inconsistent. There is concern that studies based on samples collected in late pregnancy may be confounded by kidney function but studies of the relation between pregnancy-induced changes in PFAS and kidney function are lacking. Our aims were to investigate changes in serum concentrations of perfluorononanoic acid (PFNA), perfluorooctanoic acid (PFOA), perfluorooctane sulfonate (PFOS) and perfluorohexane sulfonate (PFHxS) from early to late pregnancy and to explore relations to changes in glomerular filtration rate (GFR) and glomerular pore size.

**Methods:**

We conducted the study in a cohort of 73 pregnancies of normal-weight Swedish women without gestational diabetes and preeclampsia, enrolled 2009–2014. Blood was collected in median weeks 11 and 36, respectively, and analysed PFAS using liquid chromatography-tandem-mass-spectrometry. We estimated GFR based on creatinine and cystatin C and used the ratio eGFR_cystatin C_/eGFR_creatinine_ to indicate glomerular pore size. We used Wilcoxon signed-rank test to compare early and late measures and partial Spearman rank correlations to explore relations between changes in PFAS and kidney function.

**Results:**

Median concentrations of PFNA, PFOA and PFOS decreased by 15–21% but changes were uncorrelated to changes in kidney function (partial *R* = − 0.06–0.11). The observed increase in median PFHxS concentration of 69% was likely an artefact of systematic measurement error caused by coeluting endogenous inferences.

**Conclusions:**

Serum concentrations of PFNA, PFOA and PFOS decrease during pregnancy but the magnitudes of change are unrelated to parallel changes in eGFR and glomerular pore size, suggesting that changes in these indicators of kidney function are not important confounders in studies of PFAS and birth weight in pregnancies without gestational diabetes and preeclampsia.

## Background

Per- and polyfluorinated substances (PFAS) are synthetic chemicals with surface-active properties that have been used extensively for industrial purposes and in consumer products since the 1940’s. Today, exposure is ubiquitous and legacy PFAS, i.e. perfluorooctanoic acid (PFOA), perfluorooctane sulfonate (PFOS) and perfluorohexane sulfonate (PFHxS), as well as other PFAS are detected in the serum of close to all pregnant women [1–3]. PFAS are endocrine disrupting chemicals with biological half-lives of up to several years [4]. PFAS cross the placental barrier [5–7] and there is concern that *in utero* exposure might hamper the growth and development of the fetus.

Earlier systematic reviews and meta-analyses suggested slight inverse associations between maternal serum concentrations of PFAS and birth weight that were regarded as moderately likely to reflect a causal relationship [8–10]. The body of literature has expanded over time and, in 2018, Steenland and co-workers performed an updated meta-analysis adding 9 new studies [11]. They found that the time of blood sampling affected whether or not an association was found: there was no effect when sampling was performed early in pregnancy or shortly before conception but an inverse relationship was observed when sampling was performed in late pregnancy.

Maternal serum concentrations of many substances vary due to normal physiological changes that take place during pregnancy. Sparse scientific evidence suggests that serum concentrations of PFOA, PFOS and perfluorononanoic acid (PFNA) decrease by 11 to 33% from early to late pregnancy whereas the behaviour of PFHxS is less clear [7, 12, 13]. The observed decline has been attributed to a dilution effect following plasma volume expansion and to transfer of part of the maternal body burden to the child. A further explanation may be offered by pregnancy-induced changes in kidney function [14, 15].

Renal mechanisms affect PFAS serum concentrations through excretion via glomerular filtration and reabsorption in tubuli [16]. The glomerular filtration rate (GFR), i.e. the volume of the filtered primary urine per time unit, increases dramatically during pregnancy and is 50% higher in the third trimester compared with non-pregnant levels [17, 18] and increased GFR might imply lower serum concentrations of PFAS [15]. In humans, tubular reabsorption of PFAS is extremely effective [19] and a critical process in determining renal clearance [16]. Reabsorption is an active process governed by apical membrane transporters, in particular organic anion transporter 4 (OAT4) [16, 20], whose activity may also be altered during pregnancy [21].

Inverse cross-sectional associations between serum PFOA concentrations and estimated GFR (eGFR) have been reported in non-pregnant populations [22, 23] and scrutiny of the direction of causation suggests that the PFOA concentration is partly a consequence of kidney function [23]. Further, there is indication that the distribution of PFAS over stages of glomerular function, from normal to kidney failure, is inverted U-shaped [24]. GFR is positively associated with birth weight [25]. Thus, an inadequate rise in GFR during pregnancy may be associated with lower birth weight as well as with higher serum concentrations of PFAS. Part of the epidemiological associations between prenatal PFAS exposure and lower birth weight may consequently originate from confounding by GFR rather than from an effect of PFAS. Indeed, confounding by GFR is likely most important when blood is collected late in pregnancy when the changes in GFR should have occurred [11, 14]. This theory is in agreement with the findings of Steenland et al. [11] However, despite the potential role of GFR as a confounder of associations between PFAS and birth weight, the relation between pregnancy-induced changes in GFR and serum concentrations of PFAS has hitherto not been explored.

Plasma or serum creatinine (molecular weight 113 Da) is the most widely used marker to estimate GFR in clinical settings. It is far from ideal as the concentration of circulating creatinine is influenced by non-renal factors such as age, sex, muscle mass and diet. Cystatin C (13.3 kDa) is an alternative marker that is unaffected by dietary protein intake and substantially less sensitive to the impact of muscle mass [26]. The use of cystatin C increases worldwide although it is not yet fully implemented in clinical practice and research.

During normal pregnancy, several studies have shown that the plasma concentration of cystatin C is stable until the second trimester but increases in the third [27–29], whereas Babay et al. [30] reported lower cystatin C concentration in the second trimester as compared with the third. Although the pathophysiology is not completely known, the altered plasma concentrations are inferred to reflect a highly dynamic filtration process during pregnancy with increased filtration of low-mass molecules and decreased filtration of medium-sized molecules (10–30 kDa) [28]. The underlying mechanism is thought to be a reduction of the functional pore size in the glomerular membranes so that medium-sized molecules are selectively retained in the blood [31, 32]. This phenomenon was first observed in pregnant women and is termed ‘Shrunken pore syndrome’. In this context, it should be noted that despite the physiological changes cystatin C is still a reliable marker of GFR, measured as iohexol clearance, in the third trimester [27].

The Shrunken pore syndrome is operationally defined as eGFR_cystatin C_/eGFR_creatinine_ ≤ 0.6 [31], sometimes ≤0.7 [33]. Seniors with this syndrome show decreased filtration of molecules with a mass of 3.5 to 66.5 kDa, although molecules with a mass as low as 200 Da may be retained [32]. PFAS in serum is mainly bound to proteins, dominated by albumin, with molecular masses above those affected by shrunken pores. However, the molecular masses of PFHxS, PFOS, PFOA and PFNA are 400 to 500 Da and we hypothesize that reduced glomerular pore size in late pregnancy might affect the unbound fraction.

Our aims were to 1) investigate how serum concentrations of PFNA, PFOA, PFOS and PFHxS change from early to late pregnancy and 2) explore whether these changes could be attributed to parallel changes in kidney function, measured as eGFR (creatinine and cystatin C based) and glomerular pore size (eGFR_cystatin C_/eGFR_creatinine_).

## Methods

### Study design

We performed the study in a cohort of pregnant women that constituted the control group of the Pregnancy Obesity Nutrition and Child Health study (PONCH). The original aim of PONCH was primarily to assess the impact of a longitudinal dietary intervention on gestational weight gain and body composition and the subsequent health of the child but analysis of some environmental contaminants were also included [34, 35].

### Setting

Pregnant women from the general population in the Västra Götaland region of Sweden were enrolled between 2009 and 2014. Participants were recruited in early pregnancy through antenatal clinics, postings at public billboards or advertisement on a website for pregnant women. Study visits in the first and third trimesters were arranged at the Sahlgrenska University Hospital.

### Participants

Normal-weight women (body mass index, BMI, 18.5–24.9) aged 20 to 45 years at the time of recruitment were eligible for inclusion in the present study. Women of non-European descent or with self-reported diabetes, users of neuroleptic drugs, vegetarians and vegans were excluded. Furthermore, women with a multiple pregnancy and women who developed gestational diabetes or pre-eclampsia were excluded. In total, we included 73 women who had given blood samples in both early and late pregnancy. Information on PFAS levels in the municipal drinking water at the home address was obtained for most women through linkage to drinking water sources without indication of elevated levels.

### Collection of blood samples

Blood sampling was performed in gestation weeks 8–12 (median 11) and 35–37 (median 36), respectively. Women attended study visits in the morning after an overnight fast. Venous blood was collected using vacutainer tubes. After centrifugation, serum was aliquoted using glass pipettes washed in acetone, and stored at − 20 °C until analysis. Height was measured to the nearest centimetre. Weight was measured using a Tanita BWB-627-A electronic scale.

### Laboratory methods

#### PFAS

The analyses of total (i.e. free and protein bound) PFNA, PFOA, PFOS and PFHxS were performed in 2014 at the laboratory of Occupational and Environmental Medicine, Lund University, Sweden, using liquid chromatography-tandem-mass-spectrometry (LC/MS/MS). The method is described in detail by Lindh et al. [36]. In brief, aliquots of 100 μL serum were added with labeled internal standards for all compounds. The proteins were precipitated with acetonitrile and vigorously shaken. The samples were analyzed using a LC (UFLCXR, Shimadzu Corporation, Kyoto, Japan) connected to the MS/MS (QTRAP 5500, AB Sciex, Foster City, CA, USA). In each analytical batch, calibration standards, two homemade quality control samples and chemical blank samples were included. The samples were analyzed in duplicates and in a randomized order. The limits of detection were 0.01 ng/mL for PFNA, 0.02 ng/mL for PFOA, 0.06 ng/mL for PFOS and 0.03 ng/mL for PFHxS. The coefficient of variation of the quality control samples were for PFNA 4% at 1 ng/mL and 5% at 3 ng/mL, for PFOA 6% at 3 ng/mL and 7% at 4 ng/mL, for PFOS 7% at 6 ng/mL and 6% at 10 ng/mL and for PFHxS 17% at 2 ng/mL and 11% at 4 ng/mL. The laboratory participates in an interlaboratory exercise for PFOS and PFOA (Erlangen-Nuremberg, Germany).

#### Creatinine and cystatin C

In 2019, all samples were analyzed for creatinine, cystatin C and sodium on a Cobas 701 instrument (Roche Diagnostics, Basel, Switzerland). Sodium was included in the panel as a control to exclude evaporation during storage or insufficient mixture of the thawed sample. No such indications were observed.

### eGFR and glomerular pore size

We estimated GFR based on creatinine as well as cystatin C and applied formulas used in the US and in our laboratory (Supplemental Tables 1 and 2) [37–40]. The mean value of eGFR_creatinine_ and eGFR_cystatin C_ for each sample was also calculated as it is considered the most accurate estimate compared with invasive gold-standard methods [41].

The ratio between eGFR_cystatin C_ and eGFR_creatinine_ provides an estimate of glomerular pore size. The Shrunken pore syndrome is defined as eGFR_cystatin C_/eGFR_creatinine_ ≤ 0.6 [31]. We calculated the ratios between the equation pairs CAPA – LMrev and CKD-EPI_c__ystatin C_ – CKD-EPI_creatinine_, respectively.

### Statistical analyses

All information was available for all study participants. Data was not normally distributed and we therefore used medians together with 5th and 95th percentiles for descriptive statistics and the Wilcoxon signed-rank test for paired samples to compare early and late measures of PFAS concentrations and kidney function parameters.

We used Spearman rank correlations to assess cross-sectional associations between different PFAS and between PFAS and kidney function parameters. Further, we estimated associations between early and late concentrations of individual PFAS as well as between parallel changes in PFAS and kidney function parameters. For the latter, we explored unadjusted as well as partial correlations adjusting for the number of days that elapsed between samplings and the pregnancy-induced change in BMI as these variables may influence the changes that occur in both PFAS concentrations and kidney function parameters and thus may confound the associations.

We performed all statistical analyses in SAS version 9.4 (SAS Institute, Cary, NC).

## Results

The 73 study participants were normal-weight women and 60% were nullipara (Table 1).

**Table 1:** Characteristics of the 73 study participants at samplings in early and late pregnancy

	Median	5th and 95th percentiles	n (%)
Age in early pregnancy (years)	31	25; 37	
Early-pregnancy BMI (kg/m^2^)	22.0	19.8; 24.3	
Late-pregnancy BMI (kg/m^2^)	25.9	23.5; 29.2	
Parity			
0			44 (60)
1			21 (29)
2			8 (11)
Gestational age at early sampling (days)	82	64; 94	
Gestational age at late sampling (days)	252	243; 263	

The serum concentrations of PFAS were above the limit of detection in all samples. The median concentrations (ng/mL) at the early-pregnancy sampling were 0.7 for PFNA, 1.8 for PFOA, 5.6 for PFOS and 1.9 for PFHxS (Table 2). The median serum concentration of PFNA, PFOA and PFOS decreased from early to late pregnancy by 21, 19 and 15%, respectively. Unexpectedly, the concentration of PFHxS increased by 69%.

**Table 2:** Early and late pregnancy serum concentrations (ng/mL) of PFAS together with the relative changes that occurred during pregnancy

	Early		Late			Change during pregnancy, (%)	
	Median	5th and 95th percentiles	Median	5th and 95th percentiles	*p*-value	Median	5th and 95th percentiles
PFNA	0.7	0.4; 1.3	0.5	0.3; 1.1	< 0.001	−21	−37; − 1
PFOA	1.8	0.8; 4.4	1.5	0.7; 3.1	< 0.001	−19	−36; 7
PFOS	5.6	2.6; 11.5	4.8	1.9; 8.4	< 0.001	−15	−33; 9
PFHxS	1.9	1.3; 3.3	3.3	1.6; 5.8	< 0.001	69	3; 175

PFNA, PFOA and PFOS were highly correlated with each other with similar coefficients in early as in late pregnancy (Table 3). In contrast, correlation coefficients between PFHxS and the other substances were markedly smaller, especially in late pregnancy. Cross-sectional correlations between PFAS concentrations and kidney function parameters were consistently weak and non-significant (Supplemental Tables 3 and 4). There were strong positive correlations between early and late pregnancy measures of individual PFAS, although the coefficient was slightly smaller for PFHxS.

**Table 3:**
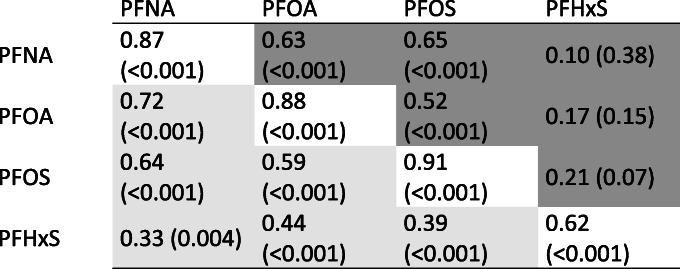
Spearman rank correlations (*p*-value) between serum concentrations of PFAS. Correlations between early and late measures of the same compound are shown on the diagonal. The lower-left off diagonal shows correlations between different compounds in early pregnancy whereas the upper-right off diagonal shows correlations between different compounds in late pregnancy

We observed a substantial increase in the plasma cystatin C concentration from early to late pregnancy (Table 4). There was also a very small increase in creatinine levels. Consequently, cystatin C-based estimating equations indicated a decrease in eGFR by 40%, whereas creatinine-based equations resulted in a neglectable reduction of eGFR. The mean of creatinine and cystatin C estimating equations suggested a decrease of approximately 20%.

**Table 4:** Kidney function parameters in early and late pregnancy together with the relative changes that occurred during pregnancy

	Early		Late			Change during pregnancy, (%)	
	Median	5th and 95th percentiles	Median	5th and 95th percentiles	*p*-value	Median	5th and 95th percentiles
*Marker*							
Plasma creatinine (μmol/L)	57	45; 67	58	46; 72	0.004	4	−11; 23
Plasma cystatin C (mg/L)	0.63	0.55; 0.79	1.05	0.77; 1.38	< 0.001	65	26; 116
*eGFR (mL/min/1.73m*^*2*^*)*							
LMrev	104	90; 120	102	86; 118	0.004	− 2	−12; 8
CKD-EPI_creatinine_	119	102; 131	119	96; 130	0.003	−1	−16; 5
CAPA	136	104; 160	76	54; 108	< 0.001	−44	−59; −23
CKD-EPI_cystatin C_	123	109; 133	76	53; 112	< 0.001	−38	− 57; −12
Mean of LMrev and CAPA	120	101; 140	89	72; 112	< 0.001	−24	−35; −13
Mean of CKD-EPI_creatinine_ and CKD-EPI_cystatin C_	121	110; 130	97	78; 118	< 0.001	−20	−31; −7
*eGFR*_*cystatin C*_*/eGFR*_*creatinine*_^a^							
CAPA/LMrev	1.3	1.0; 1.5	0.7	0.5; 1.0	< 0.001	−40	−60; −24
CKD-EPI_cystatin C_/CKD-EPI_creatinine_	1.0	0.9; 1.1	0.7	0.5; 0.9	< 0.001	−35	−55; −13

^a^eGFR_cystatin C_/eGFR_creatinine_ ≤ 0.6 defines Shrunken pore syndrome

The ratio between eGFR_cystatin C_/eGFR_creatinine_, taken to represent glomerular pore size, decreased during pregnancy. Shrunken pore syndrome was not observed in early pregnancy. However, in the third trimester 8 participants (11%) met the criteria with the CAPA and LMrev equations and 23 (32%) with the CKD-EPI equations.

We did not find evidence of association between pregnancy-induced changes of PFAS concentrations and parallel changes in any of the kidney function parameters in women with normal renal function (Table 5). The unadjusted correlations differed only marginally from the partial correlations (Supplemental Table 5).

**Table 5:** Partial Spearman rank correlations (p-value), adjusting for number of days between samplings and change in BMI, between pregnancy-induced changes in serum concentrations of PFAS and parallel changes in kidney function parameters

Kidney function parameter	ΔPFNA	ΔPFOA	ΔPFOS	ΔPFHxS
*eGFR*				
ΔLMrev	0.03 (0.82)	0.002 (0.99)	0.02 (0.85)	−0.20 (0.10)
ΔCKD-EPI_creatinine_	0.04 (0.74)	0.03 (0.83)	0.02 (0.87)	−0.20 (0.10)
ΔCAPA	0.09 (0.47)	0.06 (0.64)	−0.04 (0.73)	−0.11 (0.36)
ΔCKD-EPI_cystatin C_	0.10 (0.39)	0.03 (0.83)	−0.05 (0.66)	−0.15 (0.23)
ΔMean of LMrev and CAPA	0.09 (0.45)	0.04 (0.76)	−0.04 (0.76)	−0.14 (0.23)
ΔMean of CKD-EPI_creatinine_ and CKD-EPI_cystatin C_	0.09 (0.47)	0.002 (0.98)	−0.05 (0.66)	− 0.22 (0.07)
*eGFR*_*cystatin C*_*/eGFR*_*creatinine*_				
ΔCAPA/LMrev	0.11 (0.35)	0.09 (0.47)	−0.05 (0.68)	−0.09 (0.46)
ΔCKD-EPI_cystatin C_/CKD-EPI_creatinine_	0.08 (0.49)	−0.003 (0.98)	−0.06 (0.63)	− 0.10 (0.39)

## Discussion

Median serum concentrations of PFNA, PFOA and PFOS decreased by 15 to 21% from early to late pregnancy, whereas that of PFHxS increased by 69%. The findings for PFNA, PFOA and PFOS are well in line with those of another Swedish study of 19 primiparous women, where mean serum concentrations decreased by 33, 16 and 11%, respectively, between the first and third trimester (PFHxS not reported) [13]. A study of 71 US women found that the geometric means of PFNA, PFOA and PFOS decreased by 26 to 43% between gestational week 16 and delivery, whereas PFHxS was unaltered [12]. The decrements were slightly larger than those in our study but the discrepancy can likely be explained by blood loss during delivery.

Concentrations of PFHxS in late pregnancy were substantially higher than expected and we found a dramatic increase during pregnancy. We interpret this finding as an artefact of systematic measurement error rather than a true increase. In 2014 when the PFAS-analyses were conducted, the most sensitive tandem mass spectrometric transition (*m/z* 399/80) was commonly used. However, coeluting endogenous steroid sulfates have been suggested to share common fragmentation pathways with PFHxS which may result in overestimation using this transition [42]. Inferences from isopregnanalone, a precursor of progesterone, and pregnandiol, a degradation product of progesterone, are of particular interest in samples from pregnant women. The progesterone level rises during pregnancy, implying that PFHxS measurements in late pregnancy are prone to systematic measurement error. Our finding of lower correlations between PFHxS and the other PFAS in late pregnancy compared with early is consistent with this theory. Unfortunately, we did not have serum left to reanalyse samples for PFHxS with another transition. In this context, it should be noted that measures of PFNA, PFOA and PFOS are unaffected by inferences by progesterone metabolites. Inferences in quantification are of greatest concern in populations with low exposure levels where the ratio PFHxS:progesterone metabolites is smaller. Pregnancy is a frequently studied exposure window with respect to PFAS and studies are often performed in populations with background levels of exposure. We therefore call for scrutiny of constituents of the signals currently being interpreted as PFHxS so that the validity of measurements in different stages of pregnancy can be assessed.

Decreasing PFAS concentrations during pregnancy may well result from dilution [13], placental transfer [5–7] and upregulated GFR [14, 15]. According to formula 2a in Thompson et al. [43], the body weight change alone would cause a 15 to 20% decrease in serum concentrations under the assumptions that 1) the population is exposed to background levels that can be considered constant between early and late pregnancy and 2) volumes of distribution and elimination rates are the same in pregnant women as in the non-pregnant population. This is well in line with our findings and suggests that the changes might primarily be driven by weight gain. The assumption of background-level of exposure probably holds in our case, but both volumes of distribution and elimination rates may change during pregnancy because of the increased blood volume. Pregnancy-specific estimates of volumes of distribution and elimination rates are needed to better understand the toxicokinetics of PFAS in pregnant women.

We did not observe any increase in eGFR irrespective of whether we used creatinine or cystatin C-based estimating equations. On the contrary to what was expected, the creatinine concentration was numerically consistent between early and late pregnancy. Others have shown a reduction in creatinine concentrations of pregnant women compared with their pre-pregnancy level or  compared with non-pregnant women [27, 28]. The findings of Kristensen et al. [28] do however suggest that the reduction occurs already in weeks 6 to 13 of pregnancy. Thus, we might not have been able to capture the drop when the median week for our early sampling was 11.

In contrast, we found a marked decrease in cystatin C-based eGFR in late pregnancy driven by a substantially increased plasma concentration. Similar observations were reported by Strevens et al. [27] who inferred that the increase of serum cystatin C during pregnancy most likely was caused by an altered filtration process rather than an increased production rate. Retention of cystatin C in the blood in late pregnancy is in agreement with a pathophysiological model pertaining to reduced glomerular pore size.

The degree of albumin binding varies between different compounds [44]. Sulfonates exhibit increased affinity for bovine serum albumin relative to their equivalent chain-length carboxylate and the affinity of perfluoroalkyl carboxylates decreases from the C_8_ to C_12_ [45]. Stronger binding affinity of PFOS implicates a smaller free fraction available for renal clearance compared with PFNA and PFOA and thus less excretion, which also seemed to be the case. During pregnancy, the concentration of albumin is reduced through plasma volume expansion and increased extravascular volume [28] and there is a slightly increase in urinary excretion [46]. These processes may contribute to decreased serum concentrations of the albumin-bound fraction of PFAS, although primarily not mediated by GFR.

In contrast to protein-bound PFAS, the free fraction and the fraction bound to small proteins are readily available for renal clearance. We hypothesized that the pregnancy-induced change in PFAS concentrations might be related to parallel changes in renal filtration but our findings did not support this theory. On the contrary, there were no correlations between changes in PFAS concentrations and changes in neither eGFR nor glomerular pore size. Our findings imply that pregnancy-induced changes in glomerular filtration do not confound associations between PFAS and birth weight.

One possible explanation for the lack of association might be that the free fraction is very small so that the increased serum concentration caused by retention is negligible compared to the dilution-related decrease. Additionally, even though the glomerular pore size did decrease, pores might still be large enough to allow excretion of PFAS. It should be noted that these explanations are speculative and that other mechanisms should be explored. During pregnancy, the rising progesterone level downregulates the activity of OAT4 [21]. It is plausible that this downregulation can contribute to increased excretion of PFAS and consequently lower serum concentrations as pregnancy progresses.

The study was based on a cohort of normal-weight women without gestational diabetes and preeclampsia and confirmed background levels of exposure. Serum concentrations of PFNA, PFOA and PFOS in early pregnancy corresponded well with those of other pregnant Swedish populations at the time, i.e. the SELMA study where samplings were performed 2007–2010 and PFAS were analysed at the same laboratory [47]. Thus, the generalisability of our findings to the general population of pregnant women should be high but they might not be applicable to preeclamptic pregnancies where cystatin C retention in blood is more pronounced [27].

Currently available estimating equations for eGFR have been developed in non-pregnant populations and tend to underestimate GFR in pregnant women because physiological changes affect the association between plasma concentration of creatinine and GFR [48]. Thus, eGFR may suffer from systematic measurement error although we judge it to be of less concern in the present study as our analyses concern correlations between *changes* in PFAS and eGFR rather than the absolute figures *per se*.

## Conclusions

Serum concentrations of PFNA, PFOA and PFOS declined by approximately 20% during pregnancy, partly but not fully explained by plasma volume expansion. Correlations between early and late concentrations were high. We did not find any association between changes in PFAS concentrations and parallel changes in neither eGFR nor glomerular pore size in pregnancies without gestational diabetes and preeclampsia. Thus, although PFAS concentrations changed markedly between early and late gestation, pregnancy-induced changes in the studied aspects of kidney function did not affect the magnitude of change. The lack of associations suggests that pregnancy-induced changes in GFR and glomerular pore size are not important confounders in epidemiological studies of maternal PFAS concentrations and birth weight, at least in pregnancies without gestational diabetes and preeclampsia. Moreover, our results highlight the importance of potential systematic measurement error in the quantification of PFHxS in pregnant women with general population exposure because of inferences from progesterone metabolites.

## Supplementary information

**Additional file 1: Table S1.** Formulas used to estimate relative GFR (mL/min/1.73 m^2^) by The Chronic Kidney Disease Epidemiology Collaboration. **Table S2.** Formulas used to estimate relative GFR (mL/min/1.73 m^2^) by our laboratory. **Table S3.** Spearman rank correlations (*p*-value) between serum concentrations of PFAS and kidney function parameters in early pregnancy. **Table S4.** Spearman rank correlations (*p*-value) between serum concentrations of PFAS and kidney function parameters in late pregnancy. **Table S5.** Unadjusted Spearman rank correlations (*p*-value) between pregnancy-induced changes in serum concentrations of PFAS and parallel changes in kidney function parameters.

## Data Availability

Data contains sensitive personal information and cannot be made publicly available for legal reasons (GDPR). However, it can be accessed by other researchers after renewed ethical vetting. Any data inquiries are referred to the corresponding author.

## Abbreviations

PFAS: Perfluoroalkyl substances
PFNA: Perfluorononanoic acid
PFOA: Perfluorooctanoic acid
PFOS: Perfluorooctane sulfonate
PFHxS: Perfluorohexane sulfonate
GFR: Glomerular filtration rate
eGFR: Estimated glomerular filtration rate
BMI: Body mass index
OAT4: Organic anion transporter 4
