# Supplementary material for: Pregnancy-induced changes in serum concentrations of perfluoroalkyl substances and the influence of kidney function
Source: Environ Health. 2020 Jul 8;19:80. doi: 10.1186/s12940-020-00626-6 (PMC7346349; doi:10.1186/s12940-020-00626-6)
Supplement: Supplementary file 1 — Additional file 1: Table S1. Formulas used to estimate relative GFR (mL/min/1.73 m2) by The Chronic Kidney Disease Epidemiology Collaboration. Table S2. Formulas used to estimate relative GFR (mL/min/1.73 m2) by our laboratory. Table S3. Spearman rank correlations (p-value) between serum concentrations of PFAS and kidney function parameters in early pregnancy. Table S4. Spearman rank correlations (p-value) between serum concentrations of PFAS and kidney function parameters in late pregnancy. Table S5. Unadjusted Spearman rank correlations (p-value) between pregnancy-induced changes in serum concentrations of PFAS and parallel changes in kidney function parameters. [file 12940_2020_626_MOESM1_ESM.docx]

# Supplementary information

*Supplementary Table 1. Formulas used to estimate relative GFR (mL/min/1.73 m^2^) by The Chronic Kidney Disease Epidemiology Collaboration.*

| Formula | Biomarker | Estimating equation |
| --- | --- | --- |
| CKD-EPI_creatinine_ | Creatinine (μmol/L) | *Females with creatinine ≤62:*  144 × (creatinine/62)^-0.329^ × 0.993^age^  *Females with creatinine >62:*  144 × (creatinine/62)^-1.209^ × 0.993^age^ |
| CKD-EPI_cystatin C_ | Cystatin C (mg/L) | *Females with cystatin C ≤0.8:*  133 × (cystatin C/0.8)^-0.499^ × 0.996^age^ × 0.932  *Females with cystatin C >0.8:*  133 × (cystatin C/0.8)^-1.328^ × 0.996^age^ × 0.932 |

*Supplementary Table 2. Formulas used to estimate relative GFR (mL/min/1.73 m^2^) by our laboratory.*

| Formula | Biomarker | Estimating equation |
| --- | --- | --- |
| LMrev | Creatinine (μmol/L) | *Females with creatinine <150:*  2.50 + 0.0121 × (150 – creatinine) |
| CAPA | Cystatin C (mg/L) | 130 × cystatin C^-1.069^ × age^-0.117^ – 7 |

*Supplementary Table 3. Spearman rank correlations (p-value) between serum concentrations of PFAS and kidney function parameters in early pregnancy.*

| Kidney function parameter | PFNA | PFOA | PFOS | PFHxS |
| --- | --- | --- | --- | --- |
| *eGFR* |  |  |  |  |
| LMrev | -0.11 (0.35) | 0.13 (0.27) | -0.07 (0.58) | 0.00 (1.00) |
| CKD-EPI_creatinine_ | -0.16 (0.18) | 0.16 (0.18) | -0.08 (0.49) | 0.05 (0.66) |
| CAPA | -0.07 (0.57) | 0.11 (0.36) | 0.08 (0.51) | -0.07 (0.55) |
| CKD-EPI_cystatin C_ | -0.11 (0.36) | 0.13 (0.28) | 0.07 (0.57) | -0.05 (0.69) |
| Mean of LMrev and CAPA | -0.13 (0.29) | 0.12 (0.33) | 0.01 (0.91) | -0.03 (0.77) |
| Mean of CKD-EPI_creatinine_ and CKD-  EPI_cystatin C_ | -0.19 (0.11) | 0.14 (0.23) | -0.02 (0.89) | 0.03 (0.83) |
| *eGFR_cystatin C_/eGFR_creatinine_* |  |  |  |  |
| CAPA/LMrev | -0.01 (0.95) | 0.02 (0.89) | 0.16 (0.18) | -0.11 (0.35) |
| CKD-EPI_cystatin C_/CKD-EPI_creatinine_ | 0.05 (0.66) | -0.08 (0.52) | 0.15 (0.20) | -0.17 (0.16) |

*Supplementary Table 4. Spearman rank correlations (p-value) between serum concentrations of PFAS and kidney function parameters in late pregnancy.*

| Kidney function parameter | PFNA | PFOA | PFOS | PFHxS |
| --- | --- | --- | --- | --- |
| *eGFR* |  |  |  |  |
| LMrev | -0.17 (0.16) | -0.06 (0.59) | -0.04 (0.72) | -0.12 (0.33) |
| CKD-EPI_creatinine_ | -0.23 (0.05) | -0.04 (0.72) | -0.08 (0.50) | -0.08 (0.50) |
| CAPA | -0.08 (0.49) | -0.07 (0.54) | 0.08 (0.49) | -0.10 (0.42) |
| CKD-EPI_cystatin C_ | -0.08 (0.50) | -0.07 (0.54) | 0.08 (0.49) | -0.09 (0.48) |
| Mean of LMrev and CAPA | -0.13 (0.28) | -0.09 (0.46) | 0.03 (0.77) | -0.10 (0.38) |
| Mean of CKD-EPI_creatinine_ and CKD-  EPI_cystatin C_ | -0.15 (0.20) | -0.08 (0.52) | 0.01 (0.92) | -0.09 (0.44) |
| *eGFR_cystatin C_/eGFR_creatinine_* |  |  |  |  |
| CAPA/LMrev | 0.05 (0.71) | 0.01 (0.94) | 0.16 (0.19) | -0.07 (0.55) |
| CKD-EPI_cystatin C_/CKD-EPI_creatinine_ | 0.05 (0.65) | 0.01 (0.93) | 0.18 (0.12) | -0.07 (0.54) |

*Supplementary Table 5.* *Unadjusted Spearman rank correlations (p-value between pregnancy-induced changes in serum concentrations (ng/mL) of PFAS and parallel changes in kidney function parameters.*

| Kidney function parameter | ΔPFNA | ΔPFOA | ΔPFOS | ΔPFHxS |
| --- | --- | --- | --- | --- |
| *eGFR (mL/min/1.73m^2^)* |  |  |  |  |
| ΔLMrev | -0.02 (0.89) | -0.04 (0.71) | -0.03 (0.81) | -0.21 (0.08) |
| ΔCKD-EPI_creatinine_ | -0.02 (0.89) | -0.03 (0.78) | -0.04 (0.72) | -0.20 (0.08) |
| ΔCAPA | 0.10 (0.40) | 0.06 (0.61) | -0.04 (0.75) | -0.12 (0.30) |
| ΔCKD-EPI_cystatin C_ | 0.15 (0.21) | 0.06 (0.61) | -0.02 (0.84) | -0.16 (0.18) |
| ΔMean of LMrev and CAPA | 0.09 (0.45) | 0.03 (0.81) | -0.05 (0.69) | -0.16 (0.18) |
| ΔMean of CKD-EPI_creatinine_ and CKD-EPI_cystatin C_ | 0.12 (0.32) | 0.03 (0.82) | -0.04 (0.76) | -0.23 (0.05) |
| *eGFR_cystatin C_/eGFR_creatinine_* |  |  |  |  |
| ΔCAPA/LMrev | 0.12 (0.33) | 0.08 (0.49) | -0.05 (0.67) | -0.10 (0.39) |
| ΔCKD-EPI_cystatin C_/CKD-EPI_creatinine_ | 0.12 (0.32) | 0.03 (0.83) | -0.04 (0.77) | -0.12 (0.32) |
